# Assessing Potential Reservoir of Multidrug-Resistant Bacteria in the Oral Microbiota of Captive Burmese and Royal Pythons †

**DOI:** 10.3390/life15030442

**Published:** 2025-03-12

**Authors:** Inês Marques, Ana R. Pinto, José J. Martins, Nuno Alvura, Paula Telinhos, Pedro Mendes, Mónica Correia, João F. Requicha, Maria J. Saavedra

**Affiliations:** 1 Department of Veterinary Sciences, University of Trás-os-Montes e Alto Douro (UTAD), 5000-801 Vila Real, Portugal; al61924@alunos.utad.pt (I.M.); al60149@alunos.utad.pt (A.R.P.); 2 Antimicrobials, Biocides and Biofilms Unit (AB2Unit), University of Trás-os-Montes e Alto Douro (UTAD), 5000-801 Vila Real, Portugal; 3 Center for the Research and Technology of Agro-Environmental and Biological Sciences (CITAB)-Inov4Agro, University of Trás-os-Montes e Alto Douro (UTAD), 5000-801 Vila Real, Portugal; 4 Laboratory of Physiology, Department of Animal Science, University of Trás-os-Montes e Alto Douro (UTAD), 5000-801 Vila Real, Portugal; jjulio@utad.pt; 5 Animal and Veterinary Research Center (CECAV)-AL4AnimalS, University of Trás-os-Montes e Alto Douro (UTAD), 5000-801 Vila Real, Portugal; 6 Interdisciplinary Centre of Marine and Environmental Research (CIIMAR), University of Porto, 4450-208 Matosinhos, Portugal; 7 Zoo da Maia, 4470-184 Maia, Portugal; vet@zoodamaia.pt (N.A.); paulatelinhos@zoodamaia.pt (P.T.); pedromendes@zoodamaia.pt (P.M.); geral@zoodamaia.pt (M.C.)

**Keywords:** multidrug-resistant bacteria, One Health, oral cavity, microbiota, *Python bivittatus*, *Python regius*

## Abstract

The oral microbiota has a diversity of microorganisms that together maintain the homeostasis of the oral cavity. Disruptions in the balance of these microbial populations can lead to the development of oral and dental diseases. To characterize the normal oral microbiota of captive ophidians, samples were obtained from the oral cavity of eight Burmese pythons (*Python bivittatus*) and 11 royal pythons (*Python regius*), using oral swab, during routine evaluations. In the Laboratory-Antimicrobials, Biocides and Biofilms Unit at University of Trás-os-Montes e Alto Douro, the identification and antimicrobial susceptibility profile was determined using the Vitek^®^ 2 Compact automated device (bioMérieux, Marcy-l’Étoile, France). Of the 106 bacterial isolates obtained, 69% were Gram-negative species and 31% were Gram-positive bacteria. The genus *Pseudomonas* was the most frequently isolated. *Chryseobacterium indologenes*, *Escherichia coli* and *Pseudomonas aeruginosa* were the most isolated species. Antimicrobial susceptibility testing revealed that the phenotypic resistance was highest for nitrofurans (47.2%), beta-lactams (45.8%) and sulfonamides (30.6%). Twenty-one multidrug-resistant isolates (58.3%) were identified with *Acinetobacter baumannii*, *Serratia plymuthica*, *Chryseobacterium indologenes*, *Providencia rettgeri* and *Pseudomonas aeruginosa* showing the highest resistance frequencies.

## 1. Introduction

The oral cavity of snakes is colonized by a diverse microbiota of both Gram-negative and Gram-positive bacteria. Alterations in this oral microbiota can lead to the colonization of pathogenic organisms and the development of bacterial stomatitis secondary to episodes of immunodepression caused by poor dietary and environmental management, such as inappropriate temperature or nutritional deficits. In these situations, animals can show a variety of clinical signs, such as anorexia, dysphagia, gingivitis, ecchymoses, petechiae and dental exfoliation. If not treated in good time, stomatitis can develop into osteomyelitis and abscesses or cause respiratory tract disease, septicemia and death [1,2,3,4,5]. Due to the strong impact of oral disease on animal health and welfare, the oral microbiota has been the subject of several studies for the knowledge of the organisms present and because it is a niche with differentiating resistance characteristics [6].

Snakes from different regions of the world show significant changes in the oral microbiota. Factors that explain such differences include animal species, origin, wild or captive habitat, health status, type of prey and predation [1,7]. To date, a variety of pathogenic bacteria have been reported in snakes, including *Pseudomonas* spp., *Aeromonas* spp., *Morganella* spp., *Escherichia* spp., *Staphylococcus* spp., *Corynebacterium* spp., *Stenotrophomonas spp.*, *Acinetobacter* spp., *Klebsiella* spp., *Shigella* spp., *Clostridium* spp., *Salmonella* spp., *Proteus* spp. and *Providencia* spp. [8,9,10,11,12,13,14]. Many of the bacterial agents mentioned are present in the gastrointestinal tract of prey, leading to colonization of the predator’s oral microbiota. Additionally, the gastrointestinal tract of reptiles is also colonized by bacteria such as *Enterobacter* spp. and *Pseudomonas* spp., which can contaminate their own water sources, contributing to colonization of the oral cavity [15].

Bacterial stomatitis often involves an overgrowth of Gram-negative *bacteria* with *Pseudomonas* spp., *Aeromonas* spp., *Proteus* spp., *Escherichia* spp., *Salmonella* spp., *Klebsiella* spp. and *Mycobacterium* spp. being the most frequently isolated species [3,12,16,17]. Less commonly, bacteria from the genera *Fusobacterium*, *Clostridium*, *Bacteroides* and *Peptostreptococcus* have also been isolated [3].

Identifying the bacterial agents in the oral microbiota of snakes is crucial for expanding our understanding of these organisms but also for understanding the etiological agents of secondary infections that can arise from animal management. Information regarding the antimicrobial susceptibilities of snake oral bacteria is essential for supporting empirical antimicrobial therapy.

This study aimed to characterize the commensal oral Gram-negative microbiota of captive snakes and evaluate their potential as reservoirs of multidrug-resistant bacteria.

## 2. Materials and Methods

### 2.1. Animals

This study included nineteen snakes from the reptile collection at Zoo da Maia, a zoological park located in the north of Portugal, observed during routine oral examinations performed between March and April of 2023. Eight Burmese pythons (*Python bivittatus*, PB), five females and three males, with an average age of four years old (1–5 years), and 11 royal pythons (*Python regius*, PR), six females and five males with an average age of six years old (2–12 years), were selected for this study. All procedures were conducted in accordance with the European Animal Welfare Directives (Directive 98/58/CE) and the animals were handled and samples taken by three Federation of European Laboratory Animal Science Associations accredited staff.

### 2.2. Sample Processing and Isolation

Using sterile AMIES swabs (VWR, Carnaxide, Portugal), 19 samples from the oral cavity were collected under manual restraint of the animal without the need for sedation. The samples were placed in a closed tube containing a transport medium and transferred to the Antimicrobials, Biocides and Biofilms Unit (AB2Unit), Department of Veterinary Sciences at University of Trás-os-Montes e Alto Douro for bacterial isolation and identification.

Oral swabs were spread on different culture (according with the methodologies of laboratory AB2 Unit-CITAB) media such as Chromocult^®^ Coliform Agar (CCA) (Merck, Darmstadt, Germany), MacConkey Agar (Oxoid, Hampshire, UK), Baird Parker Agar (Oxoid, Hampshire, UK), Glutamate Starch Phenol Red Agar (Oxoid, Hampshire, UK), and Mannitol Salt Agar (Oxoid, Hampshire, UK), according to the manufacturer’s instructions. All plates were incubated at 36 ± 1 °C for 24 h and the shape and color of the colonies were observed.

### 2.3. Identification and Antimicrobial Susceptibility Testing of Bacterial Isolates

After obtaining pure cultures, bacterial identification and antimicrobial susceptibility testing (ID/TSA test) were performed using the automated Vitek^®^ 2 Compact system (bioMérieux, Marcy-l’Étoile, France) in accordance with the methodologies of laboratory AB2 Unit-CITAB. The identification of the microbial species was performed through a card consisting of a miniaturized system of conventional biochemical tests (Vitek^®^ 2 GP and Vitek^®^ 2 GN, bioMérieux, France). The determination of antimicrobial susceptibility was performed using cards for AST composed of multiple antibiotics. In order to obtain the ID and the AST of the bacterial isolate, the protocol established by the manufacturer and used in veterinary microbiology applications was followed.

Twenty-one antimicrobials from seven different classes were tested on Gram-negative isolates (Vitek^®^ 2 AST-GN97, bioMérieux, Craponne, France): beta-lactams: ampicillin (AMP), amoxicillin–clavulanic acid (AMC), cephalexin (CL), cephalothin (KF), cefpodoxime (CPD), cefovecin (CVN), ceftiofur (EFT), ertapenem (ETP), imipenem (IPM), meropenem (MEM); aminoglycosides: amikacin (AK), gentamicin (CN), neomycin (N); fluoroquinolones: enrofloxacin (ENR), marbofloxacin (MRB), pradofloxacin (PFX); tetracyclines: doxycycline (DO), tetracycline (TE); nitrofurans: nitrofurantoin (F); amphenicols: chloramphenicol (C); sulfonamides: trimethoprim + sulfamethoxazole (SXT).

## 3. Results

### 3.1. Bacterial Species Isolated

Following the isolation process, thirty-six Gram-negative isolates were identified, including *Chryseobacterium indologenes* (n = 6), *Escherichia coli* (n = 4), *Pseudomonas aeruginosa* (n = 4), *Pseudomonas fluorescens* (n = 2), *Pseudomonas putida* (n = 1), *Bordetella hinzii* (n = 2), *Pandoraea* spp. (n = 2), *Providencia rettgeri* (n = 2), *Acinetobacter baumannii* (n = 1), *Achromobacter denitrificans* (n = 1), *Achromobacter xylosoxidans* (n = 1), *Delftia acidovorans* (n = 1), *Enterobacter cloacae* complex (n = 1), *Lechercia adecarboxylata* (n = 1), *Pasteurella pneumotropica* (n = 1), *Proteus mirabilis* (n = 1), *Rahnella aquatilis* (n = 1), *Salmonella* group (n = 1), *Serratia plymuthica* (n = 1), *Sphingobacterium thalpophilum* (n = 1) and *Sphingomonas paucimobilis* (n = 1) (Table 1).

The genus Pseudomonas was the most identified (19.4%) with seven isolates of the species *P. aeruginosa* (n = 4), *P. fluorescens* (n = 2), and *P. putida* (n = 1). This genus was identified in 12.5% (n = 2) of the PB samples and in 20% of PR samples (*P. aeruginosa*, 5%; *P. fluorescens*, 10% and *P. putida*, 5%). Overall, 55.6% (n = 20) of the isolates were derived from PR, while 44.4% (n = 16) were isolated from the oral cavities of PB (Table 2 and Table 3).

The majority of animals in the study exhibited polymicrobial cultures except for PB7, PR3, PR6, and PR7. Several bacterial species were isolated exclusively in either PB or PR species, such as the *Salmonella* genus or *Acinetobacter baumannii* only isolated in the oral cavities of PB and PR, respectively. *Bordetella hinzii* was isolated in both python species, unlike *Escherichia coli*, which was found only in the oral cavity of PB. Bacteria from the genus *Chryseobacterium* were isolated and identified exclusively in the PR population. The three most common organisms were *Chryseobacterium indologenes*, *Escherichia coli* and *Pseudomonas aeruginosa*.

### 3.2. Antibiotic Resistance Profile

**Figure 1:**
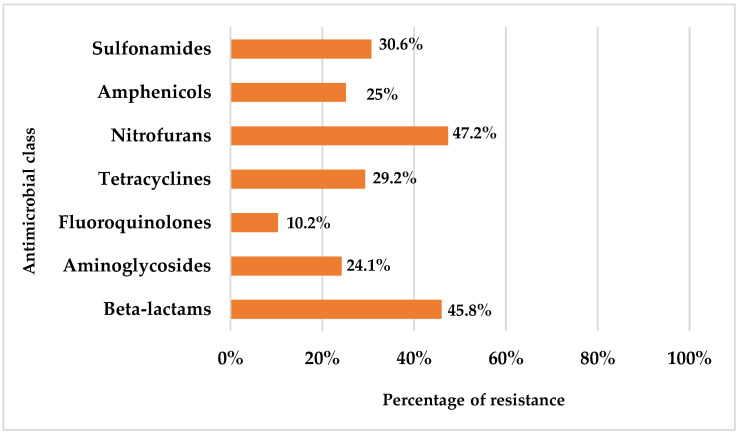
Percentage of resistance to each antimicrobial class.

The results showed high levels of phenotypic resistance, particularly to nitrofurans (47.2%), beta-lactams (45.8%) and sulfonamides (30.6%), while resistance to quinolones was comparatively lower (10.2%) (Figure 1).

**Figure 2:**
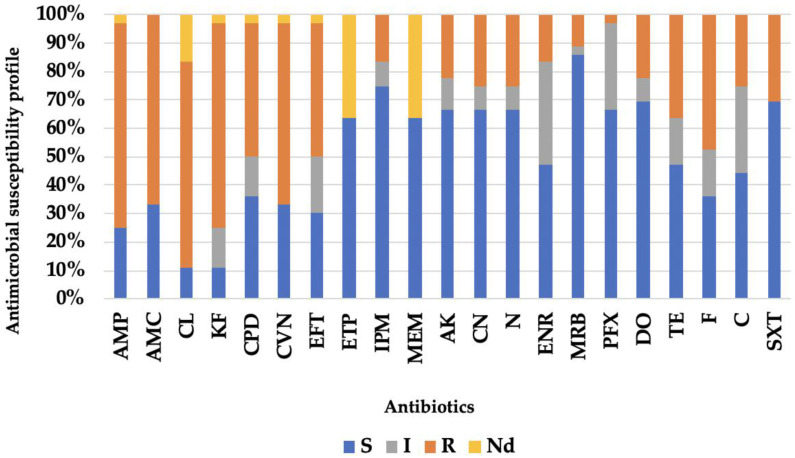
Antimicrobial susceptibility profile of the set of 36 Gram-negative isolates studied. Legend: beta-lactams: ampicillin (AMP), amoxicillin–clavulanic acid (AMC), cephalexin (CL), cephalothin (KF), cefpodoxime (CPD), cefovecin (CVN), ceftiofur (EFT), ertapenem (ETP), imipenem (IPM), meropenem (MEM); aminoglycosides: amikacin (AK), gentamicin (CN), neomycin (N); fluoroquinolones: enrofloxacin (ENR), marbofloxacin (MRB), pradofloxacin (PFX); tetracyclines: doxycycline (DO), tetracycline (TE); nitrofurans: nitrofurantoin (F); amphenicols: chloramphenicol (C); sulfonamides: trimethoprim + sulfamethoxazole (SXT); S—susceptible; I—intermediate; R—resistant; Nd—not determined.

When assessing individual resistance to each antimicrobial, beta-lactams exhibited the highest overall resistance, with ampicillin, cephalexin, and cephalothin all showing a resistance rate of 72.2%, which were followed by amoxicillin–clavulanic acid (66.7%), and ceftiofur (63.9%). Concerning aminoglycosides, resistance rates were similar for gentamicin and neomycin, with eight isolates (22.2%) resistant to amikacin, and four displaying intermediate behavior (11.1%). Within the fluoroquinolone class, marbofloxacin exhibited the highest susceptibility (86.1%; n = 31), which was followed by pradofloxacin (66.7%; n = 24) and enrofloxacin (47.2%; n = 17). Six isolates (16.7%) demonstrated resistance to enrofloxacin, four demonstrated resistance to marbofloxacin (11.1%), and only one isolate showed resistance to pradofloxacin (2.8%). The tetracycline class also exhibited enhanced antimicrobial activity with doxycycline proving more effective than tetracycline (69.4% and 47.2%, respectively). Additionally, resistance rates to trimethoprim + sulfamethoxazole, nitrofurantoin, and chloramphenicol were 47.2%, 30.6%, and 25%, respectively. Finally, it is noteworthy that 16.7% (n = 6) were identified as resistant to imipenem (Figure 2).

**Figure 3:**
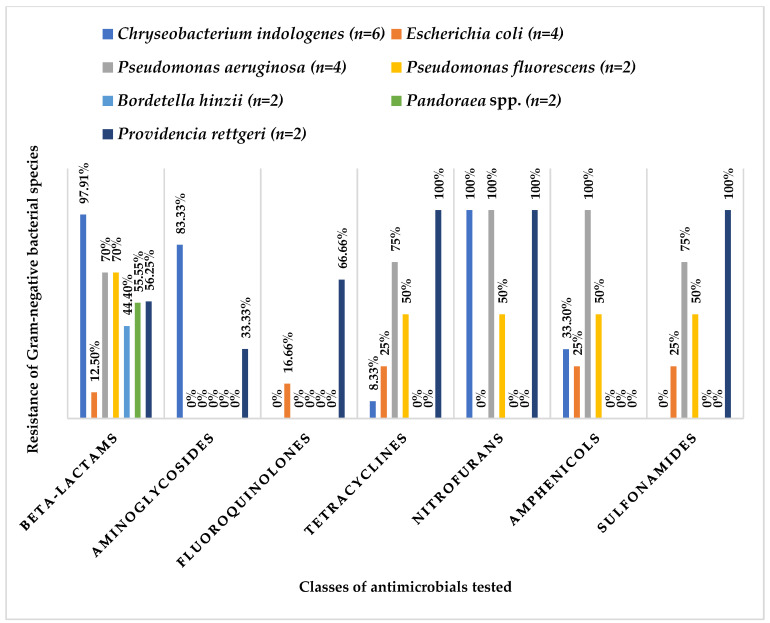
Relative frequency of resistance of Gram-negative bacterial species identified with at least two isolates in relation to the classes of antimicrobials tested.

Among the species identified in the microbiota, *Chryseobacterium indologenes*, *Pseudomonas aeruginosa* and *Providencia rettgeri* exhibited the highest resistance to the different classes of antimicrobials tested. *Acinetobacter baumannii* and *Serratia plymuthica* were the two species with the highest resistance phenotypes compared to the other species. In this study, *A. baumannii* was shown to be resistant to cephalexin, cephalothin, cefovecin, and ceftiofur, all of which belong to the beta-lactam class. It also showed a resistant phenotype to tetracycline (tetracyclines), nitrofurantoin (nitrofurans), chloramphenicol (phenicols) and trimethoprim + sulfamethoxazole (sulfonamides). However, resistance to aminoglycosides or fluoroquinolones was not observed. *S. plymuthica* demonstrated resistance to beta-lactams (ampicillin, cephalexin, cephalothin, cefovecin and imipenem), aminoglycosides (amikacin and gentamicin), fluoroquinolones (enrofloxacin and pradofloxacin), sulfonamides (trimethoprim/sulfamethoxazole) and tetracyclines (doxycycline and tetracycline) (Figure 3).

It is crucial to highlight that multidrug resistance (MDR) was observed in 21 out of 36 isolates (58,3%), indicating simultaneous resistance to three or more antimicrobial families. The MDR pattern was distributed by *Chryseobacterium indologenes* (6/6), *Escherichia coli* (1/4), *Pseudomonas aeruginosa* (4/4), *Pseudomonas putida* (1/1), *Pseudomonas fluorescens* (1/2), *Providencia rettgeri* (2/2), *Acinetobacter baumannii* (1/1), *Delftia acidovorans* (1/1), *Lechercia adecarboxylata* (1/1), *Proteus mirabilis* (1/1), *Serratia plymuthica* (1/1), and *Sphingomonas paucimobilis* (1/1). The MDR profile table shows that resistance to βLC (beta-lactams) in most isolates is often accompanied by resistance to AMN (aminoglycosides) (Table 4).

## 4. Discussion

The identification of bacteria with an MDR profile in samples from the oral cavitiy and skin of clinically healthy captivity snakes emphasized a public health problem that concerns the emergence of these microorganisms [18,19]. Due to the close contact between humans and snakes, it is highly possible that humans will be infected with multidrug-resistant strains.

This study is clinically relevant since commensal bacteria can act as opportunistic pathogens and produce infections that are difficult to treat [20].

Several bacterial species were isolated only in Burmese pythons (PB) or in royal pythons (PR), such as *Escherichia coli*, which was only identified in PB. *Escherichia coli* is a Gram-negative, facultative anaerobic bacterium that is considered a commensal organism in the intestines and present in the environment. It can act as a principal pathogen or as an opportunistic one and can be highly pathogenic. It is associated with bacterial stomatitis in reptiles [3,15,21,22].

One of the bacteria isolated exclusively in the oral cavity of PR was *Chryseobacterium indologenes*. This bacterial agent is found sporadically in soil, water, waste, food sources, animals and aquatic environments [23]. In snakes, *C. indologenes* has been reported in an oral abscess in a PR [24].

As mentioned above, the genus *Pseudomonas* was the most identified. In the study by Jho and colleagues (2011) [12], the genus *Pseudomonas* was also the most frequently isolated in the oral cavity of snakes (33% of the samples) with the identification of *P. aeruginosa* and *P. putida.* In PR, Dipineto and collaborators (2014) [25] isolated *Pseudomonas* spp. in 85% of the animals with *P. putida* found in nine animals and *P. aeruginosa* in 42 animals. Yak and colleagues (2015) [26] confirmed the abundance of the genus *Pseudomonas* in the oral cavity of reticulated pythons (*Python reticulatus*) with *P. aeruginosa* isolated in 20% of the specimens and *P. staminae* in 10%. The genus *Pseudomonas* is considered an opportunistic pathogen with a high capacity for developing resistance to antimicrobials and various virulence factors, which is why it is associated with high morbidity and mortality rates. These bacteria can be responsible for pneumonia, septicemia, skin and oral lesions such as ulcerative stomatitis [16,26].

In the present study, *Acinetobacter baumannii* and *Serratia plymuthica* were the two species with the highest resistance phenotypes compared to the other species with only one isolated each. *A. baumannii* is an opportunistic hospital pathogen that causes severe and invasive nosocomial infections associated with high mortality rates, posing a threat to public health [27,28]. The high resistance to antimicrobials shown by this bacterial genus is associated with the presence of multiple resistance mechanisms that make *A. baumannii* resistant to most beta-lactam antimicrobials, aminoglycosides, fluoroquinolones, tetracyclines, macrolides, phenicols and sulfonamides [29]. The species *S. plymuthica*, which belongs to the *Enterobacteriaceae* family, is predominantly found in water, soil, plants, and wild animals. Species from this genus are associated with various infections such as pneumonia, meningitis and urinary tract infections [30].

The bacterial species with at least two isolates that showed the highest resistance to the different classes of antimicrobials tested were *Chryseobacterium indologenes*, *Pseudomonas aeruginosa* and *Providencia rettgeri.* The genus *Chryseobacterium* is considered an opportunistic and MDR bacterial agent with the capacity to cause serious infections in neonates, pregnant women and immunosuppressed patients [24]. *C. indologenes* is intrinsically resistant to aminoglycosides, aminopenicillins, first-generation cephalosporins and aztreonam [24,31]. *P. aeruginosa* is commonly found in intensive care units and is associated with various types of infections in humans and animals, and it can also be found in soil and water. *P. aeruginosa* is resistant to various antimicrobials, including beta-lactams (3rd generation cephalosporins and carbapenems), fluoroquinolones, and aminoglycosides [32,33]. The genus *Providencia*, which was identified in two isolates (*P. rettgeri*) from PB, is also described in the oral cavity of several snakes, namely *Protobothrops mucrosquamatus*, *Bungarus multicinctus* and *Daboia siamensis* [34]. Bacteria of the *Providencia* genus are commensals of the gastrointestinal tract and have been identified in various animals as well as in water and soil [35,36].

In the treatment of bacterial stomatitis in snakes, fluoroquinolones and aminoglycosides are the common choices of first-line antibiotics while waiting for the results of AST [37,38]. Looking at the percentages of resistance in each class of antimicrobials tested on this study, nitrofurans, beta-lactams and sulfonamides presented the highest phenotypic resistance. These classes of antimicrobials belong to category D according to the EMA (European Medicines Agency) categorization and are used as first-line antimicrobials. The high phenotypic resistance observed in these classes can significantly reduce the effectiveness of antibiotic therapy, demonstrating the importance of clinical laboratory diagnosis in veterinary microbiology associated with animal health. Five isolates of the species *Chryseobacterium indologenes* and one isolate of *Serratia plymuthica* showed resistance to imipenem, which is an antibiotic used as a last resort in infections with MDR bacteria and only in hospital settings [39].

## 5. Conclusions

Wild animals play a critical role in the One Health concept, representing potential natural reservoirs of antimicrobial resistance and infection. They are also actively involved in the spread of bacteria and resistance determinants between different habitats. Emerging infectious diseases often result from the interplay between wildlife, humans, and the environment. These findings highlight the importance of personal protective equipment for a variety of roles, including animal keepers, veterinarians, and healthcare professionals.

This study has shown that snakes can be reservoirs of pathogens with zoonotic potential, carrying genes that confer resistance/multidrug resistance to highly relevant antimicrobials. The distinction between intrinsic and acquired resistance is fundamental in the study of antibiotic resistance, as it influences approaches to the treatment and control of infections. In terms of public health, acquired resistance is of greater concern, as it can spread between different bacterial species and make it difficult to treat infections, so a detailed future study would be essential. Due to the growing increase in antibiotic resistance, it is imperative to conduct studies to monitor and control antibiotic resistance, always considering a holistic approach. Antimicrobial resistance and the increase in multidrug-resistant bacteria emphasize the importance of medicine becoming increasingly aware of the correct use of antimicrobials and of raising awareness among regulatory bodies, veterinarians, animal health professionals, and guardians of the emerging problem and the risk to public health.

## Figures and Tables

**Table 1:** List of bacterial species identified in the snake samples studied (n = 19).

Identification	No of Isolates (n = 36)
*Chryseobacterium indologenes*	6
*Escherichia coli*	4
*Pseudomonas aeruginosa*	4
*Pseudomonas fluorescens*	2
*Pseudomonas putida*	1
*Bordetella hinzii*	2
*Pandoraea* spp.	2
*Providencia rettgeri*	2
*Acinetobacter baumannii*	1
*Achromobacter denitrificans*	1
*Achromobacter xylosoxidans*	1
*Delftia acidovorans*	1
*Enterobacter cloacae* complex	1
*Leclercia adecarboxylata*	1
*Pasteurella pneumotropica*	1
*Proteus mirabilis*	1
*Rahnella aquatilis*	1
*Salmonella* group	1
*Serratia plymuthica*	1
*Sphingobacterium thalpophilum*	1
*Sphingomonas paucimobilis*	1

**Table 2:** Bacterial species identified in each Burmese python (n = 8). Legend: F—female, M—male.

Animal	Sex	Age (Years)	No of Isolates	Species Identified
PB1	M	1	2	*Achromobacter xylosoxidans*
				*Bordetella hinzii*
PB2	F	4	2	*Providencia rettgeri*
				*Providencia rettgeri*
PB3	M	5	2	*Pasteurella pneumotropica*
				*Achromobacter denitrificans*
PB4	F	5	2	*Escherichia coli*
				*Escherichia coli*
PB5	F	5	3	*Escherichia coli*
				*Delftia acidovorans*
				*Salmonella* group
PB6	F	4	2	*Proteus mirabilis*
				*Escherichia coli*
PB7	F	4	1	*Pseudomonas aeruginosa*
PB8	M	4	2	*Pseudomonas aeruginosa*
				*Pseudomonas aeruginosa*

**Table 3:** Bacterial species identified in each royal python (n = 11). Legend: F—female, M—male.

Animal	Sex	Age (Years)	No of Isolates	Species Identified
PR1	F	10	2	*Chryseobacterium indologenes*
				*Chryseobacterium indologenes*
PR2	M	2	2	*Pseudomonas fluorescens*
				*Pseudomonas aeruginosa*
PR3	F	2	1	*Pandoraea* spp.
PR4	F	4	2	*Chryseobacterium indologenes*
				*Chryseobacterium indologenes*
PR5	F	12	3	*Acinetobacter baumannii*
				*Leclercia adecarboxylata*
				*Enterobacter cloacae* complex
PR6	M	2	1	*Sphingobacterium thalpophilum*
PR7	M	10	1	*Pseudomonas putida*
PR8	F	10	2	*Rahnella aquatilis*
				*Serratia plymuthica*
PR9	F	2	2	*Pseudomonas fluorescens*
				*Chryseobacterium indologenes*
PR10	M	12	2	*Pandoraea* spp.
				*Sphingomonas paucimobilis*
PR11	M	4	2	*Bordetella hinzii*
				*Chryseobacterium indologenes*

**Table 4:** Multidrug-resistant profile of oral microbiota bacteria from *Python bivittatus* and *Python regius.* Legend: βLC—beta-lactams, IMP—imipenem, AMN—aminoglycosides, FL—fluoroquinolones, TET—tetracyclines, NIT—nitrofurans, ANF—amphenicols, SUL—sulfonamides, “n”—number of isolates.

***Chryseobacterium indologenes***	**n**	***Escherichia coli***	**n**	***Pseudomonas aeruginosa***	**n**	***Pseudomonas putida***	**n**
βLC-IMP-AMN-NIT	4	βLC-FL-TET-ANF-SUL	1	βLC-TET-NIT-ANF-SUL	3	βLC-NIT-SUL	1
βLC-AMN-TET-NIT-ANF	1			βLC-TET-NIT-ANF	1		
βLC-IMP-AMN-NIT-ANF	1						
***Pseudomonas fluorescens***	**n**	***Providencia rettgeri***	**n**	***Acinetobacter baumannii***	**n**	***Delftia acidovorans***	**n**
βLC-TET-NIT-ANF-SUL	1	βLC-AMN-FL-TET-NIT-SUL	2	βLC-TET-SUL	1	βLC-AMN-FL	1
***Lechercia adecarboxylata***	**n**	***Proteus mirabilis***	**n**	***Serratia plymuthica***	**n**	***Sphingomonas paucimobilis***	**n**
AMN-FL-TET-SUL	1	βLC-TET-NIT	1	βLC- IMP-AMN-FL-TET-SUL	1	βLC-AMN-NIT	1

## Data Availability

All raw data are available from the corresponding authors upon reasonable request.

## References

[1] Padhi L., Panda S.K., Mohapatra P.P., Sahoo G. (2020). Antibiotic susceptibility of cultivable aerobic microbiota from the oral cavity of *Echis carinatus* from Odisha (India). Microb. Pathog..

[2] Mehler S.J., Bennett R.A. (2003). Oral, dental, and beak disorders of reptiles. Vet. Clin. North Am. Exot. Anim. Pract..

[3] Hedley J. (2016). Anatomy and disorders of the oral cavity of reptiles and amphibians. Vet. Clin. North Am. Exot. Anim. Pract..

[4] Mustafa S., Popova T. (2017). *Enterobacter agglomerans*—A cause of stomatitis in a snake. Tradit. Mod. Vet. Med..

[5] Grego K.F., Carvalho M.P.N., Cunha M.P.V., Knobl T., Pogliani F.C., Catão-Dias J.L., Sant´Anna S.S., Ribeiro M.S., Sellera F.P. (2017). Antimicrobial photodynamic therapy for infectious stomatitis in snakes: Clinical views and microbiological findings. Photodiagnosis Photodyn. Ther..

[6] Shek K., Tsui K., Lam K., Crow P., Ng K., Ades G., Yip K., Grioni A., Tan K., Lung D. (2009). Oral bacterial flora of the Chinese cobra (*Naja atra*) and bamboo pit viper (*Trimeresurus albolabris*) in Hong Kong SAR, China. Hong Kong Med. J..

[7] Artavia-León A., Romero-Guerrero A., Sancho-Blanco C., Rojas N., Umaña-Castro R. (2017). Diversity of aerobic bacteria isolated from oral and cloacal cavities from free-living snakes species in Costa Rica rainforest. Int. Sch. Res. Not..

[8] Babalola M.O., Balogun J.A. (2013). The ecology and potential health risk of the oral microflora of *Python regius* and *Clelia scyntalina*. Int. J. Microbiol. Res..

[9] Lukač M., Horvatek Tomić D., Mandac Z., Mihoković S., Prukner-Radovčić E., Horvatek Tomić D., ManDac Z., Mihoković S., Prukner E. (2017). Oral and cloacal aerobic bacterial and fungal flora of free-living four-lined snakes (*Elaphe quatuorlineata*) from Croatia. Veterinarski Arhiv.

[10] Dehghani R., Sharif M.R., Moniri R., Sharif A., Kashani H.H. (2016). The identification of bacterial flora in oral cavity of snakes. Comp. Clin. Pathol..

[11] Hedley J., Whitehead M.L., Munns C., Pellett S., Abou-Zahr T., Calvo Carrasco D., Wissink-Argilaga N. (2021). Antibiotic stewardship for reptiles. J. Small Anim. Pract..

[12] Jho Y.-S., Park D.-H., Lee J.-H., Cha S.-Y., Han J.S. (2011). Identification of bacteria from the oral cavity and cloaca of snakes imported from Vietnam. Lab. Anim. Res..

[13] Zancolli G., Mahsberg D., Sickel W., Keller A. (2015). Reptiles as reservoirs of bacterial infections: Real threat or methodological bias. Microb. Ecol..

[14] Gospodinova I.R., Mustafa S., Popova T.P. (2024). Microbiological analysis of laryngeal and oral swab samples from captive-bred snakes with respiratory tract infections in Bulgaria. Acta Microbiol. Bulg..

[15] Ghosh T., Biswas M.K., Roy P., Guin C. (2017). Short review of different microflora from the oral cavity of snakes. Uttar Pradesh J. Zool..

[16] Zhang F., Cheng W. (2022). The mechanism of Bbcterial resistance and potential bacteriostatic strategies. Antibiotics.

[17] Mitchell M.A., Diaz-Figueroa O. (2005). Clinical reptile gastroenterology. Vet. Clin. North Am. Exot. Anim. Pract..

[18] Silva C., Requicha J.F., Martins J.J., Duarte A., Dias I.R., Viegas C.A., Saavedra M.J. (2021). Black-and-white ruffed lemur (*Varecia variegata)* in captivity: Analysis of the oral microbiota in a one health perspective. Animals.

[19] Marques I., Alvura N., Martins J.J., Requicha J.F., Saavedra M.J. (2023). First report of *Kocuria kristinae* in the skin of a Cuban boa (*Epicrates angulifer*). Life.

[20] Pimenta J., Pinto A.R., Saavedra M.J., Cotovio M. (2023). Equine Gram-negative oral microbiota: An antimicrobial aesistances watcher?. Antibiotics.

[21] Cheng L., Cheng X., Deng M., Deng X., Du Q., Ge Y., Guo Q., He J., Jia W., Kang D., Zhou X., Li Y. (2015). Oral mucosal microbes. Atlas of Oral Microbiology: From Healthy Microflora to Disease.

[22] D’Cruze N., Bates J., Assou D., Ronfot D., Coulthard E., Segniagbeto G.H., Auliya M., Megson D., Rowntree J. (2020). A preliminary assessment of bacteria in “ranched” ball pythons (*Python regius*), Togo, West Africa. Nat. Conserv..

[23] Das P., Karade S., Kaur K., Ramamurthy R., Ranjan P. (2017). *Chryseobacterium indologenes* pneumonitis in an infant: A case report. J. Clin. Diagn. Res..

[24] Tamai I.A., Pakbin B., Kafi Z.Z., Brück W.M. (2021). Oral abscess caused by *Chryseobacterium indologenes* in ball python (*Python regius*): A case report. Antibiotics.

[25] Dipineto L., Russo T.P., Calabria M., De Rosa L., Capasso M., Menna L.F., Borrelli L., Fioretti A. (2014). Oral flora of *Python regius* kept as pets. Lett. Appl. Microbiol..

[26] Yak R., Lundin A.-C., Pin P.Y., Sebastin S.J. (2015). Oral bacterial microflora of free-living reticulated pythons (*Python reticulatus*) in Singapore. J. Herpetol. Med. Surg..

[27] Basatian-Tashkan B., Niakan M., Khaledi M., Afkhami H., Sameni F., Bakhti S., Mirnejad R. (2020). Antibiotic resistance assessment of *Acinetobacter baumannii* isolates from Tehran hospitals due to the presence of efflux pumps encoding genes (adeA and adeS genes) by molecular method. BMC Res. Notes.

[28] Kyriakidis I., Vasileiou E., Pana Z.D., Tragiannidis A. (2021). *Acinetobacter baumannii* antibiotic resistance mechanisms. Pathogens.

[29] Foti M., Giacopello C., Fisichella V., Latella G. (2013). Multidrug-resistant *Pseudomonas aeruginosa* isolates from captive reptiles. J. Exot. Pet Med..

[30] Van Houdt R., Moons P., Jansen A., Vanoirbeek K., Michiels C.W. (2005). Genotypic and phenotypic characterization of a biofilm-forming *Serratia plymuthica* isolate from a raw vegetable processing line. FEMS Microbiol. Lett..

[31] Izaguirre-Anariba D.E., Sivapalan V. (2020). *Chryseobacterium indologenes*, an emerging bacteria: A case report and review of literature. Cureus..

[32] Pachori P., Gothalwal R., Gandhi P. (2019). Emergence of antibiotic resistance *Pseudomonas aeruginosa* in intensive care unit; a critical review. Genes Dis..

[33] Roulová N., Mot’ková P., Brožková I., Pejchalová M. (2022). Antibiotic resistance of *Pseudomonas aeruginosa* isolated from hospital wastewater in the Czech Republic. J. Water Health.

[34] Chuang P.C., Lin W.H., Chen Y.C., Chien C.C., Chiu IMTsai T.S. (2022). Oral bacteria and their antibiotic susceptibilities in Taiwanese venomous snakes. Microorganisms.

[35] Benedict S., Shilton C.M. (2016). *Providencia rettgeri* septicaemia in farmed crocodiles. Microbiol. Aust..

[36] Lacerda L.E., Portela R.W. (2021). Perfil de sensibilidade a antibióticos de um isolado de *Providencia rettgeri* proveniente de equino. Rev. Ciências Médicas Biológicas.

[37] Sharina O., Jian H.S., Azlan C. (2023). Bacterial stomatitis in wild reticulated pythons (*Malayopython reticulatus*) in Malaysia. World Vet. J..

[38] Singh J., Mallik S., Nath I., Acharya A., Das S.P., Sethi S., Sahoo M. (2018). Infectious stomatitis in an Indian rock python (*Python molurus*) and its therapeutic management. J. Entomol. Zool. Stud..

[39] European Medicines Agency (2020). Categorisation of Antibiotics Used in Animals Promotes Responsible Use to Protect Public and Animal Health. https://www.ema.europa.eu/en/documents/report/categorisation-antibiotics-european-union-answer-request-european-commission-updating-scientific-advice-impact-public-health-and-animal-health-use-antibiotics-animals_en.pdf.

[40] Marques I., Pinto A.R., Alvura N., Telinhos P., Mendes P., Correia M., Martins J.J., Requicha J.F., Saavedra M.J. Oral Microbiota in captive snakes: A study in Burmese and royal pythons. Proceedings of the Book of the 31st European Veterinary Dental Forum.

